# Supplementary material for: Virtual-freezing fluorescence imaging flow cytometry
Source: Nat Commun. 2020 Mar 6;11:1162. doi: 10.1038/s41467-020-14929-2 (PMC7058616; doi:10.1038/s41467-020-14929-2)
Supplement: Supplementary file 1 — Supplementary Information [file 41467_2020_14929_MOESM1_ESM.pdf]

Supplementary Information

Virtual-freezing fluorescence imaging flow cytometry

Mikami et al.

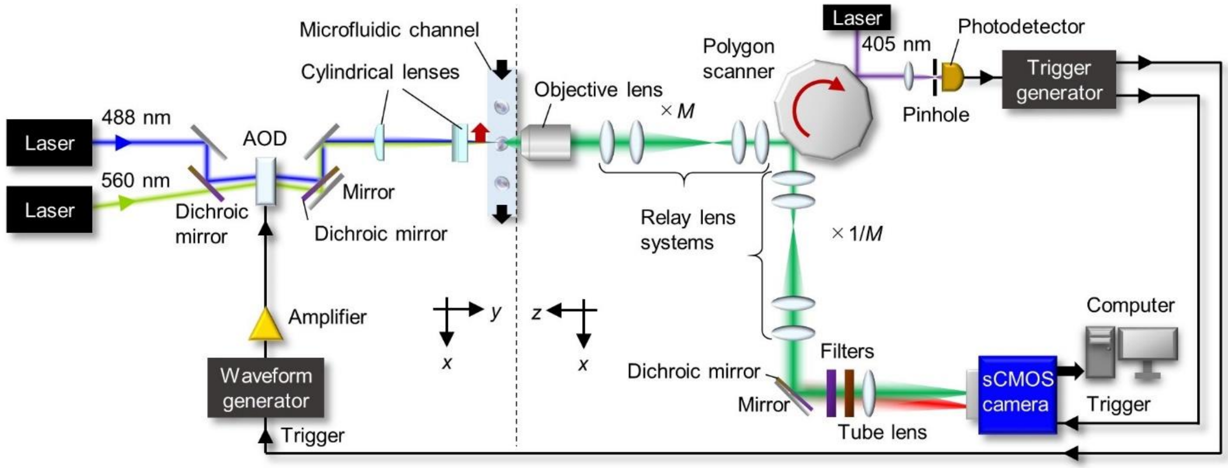

Supplementary Fig. 1 | Complete schematic of the VIFFI flow cytometer. The left hand side from the dashed line represents the light-sheet excitation system while the right hand side from the dashed line represents the imaging optical system and angle detection system for the polygon scanner. Note that the dimensional axes in this plane are different between the right and left hand sides.

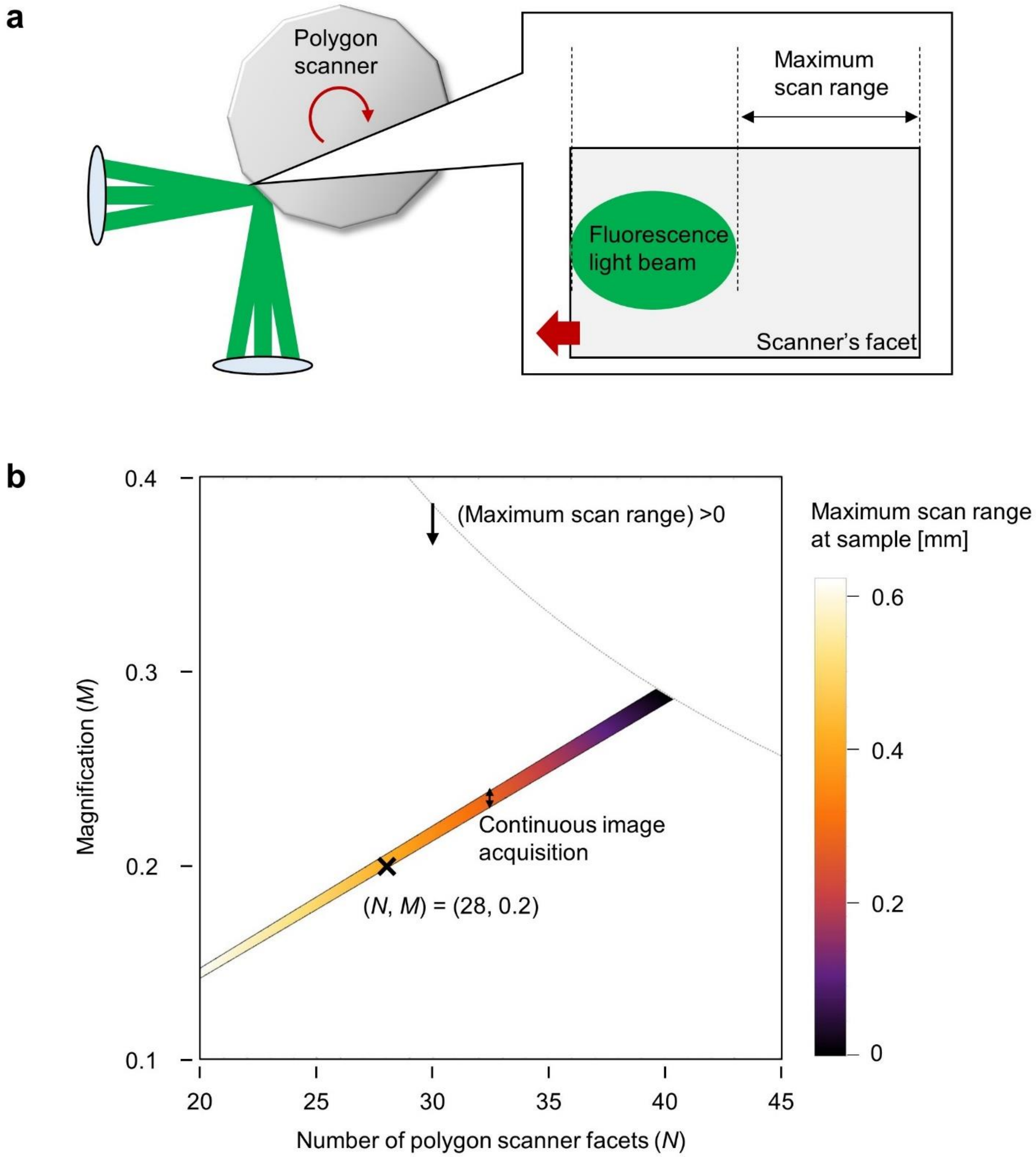

Supplementary Fig. 2 | Design of the optical system of the VIFFI flow cytometer. a, Configuration of the fluorescence light beam on a polygon scanner's facet. **b,** 2D plot of the maximum scan range in the object plane as a function of the number of polygon-scanner facets and the magnification of the first relay lens system.

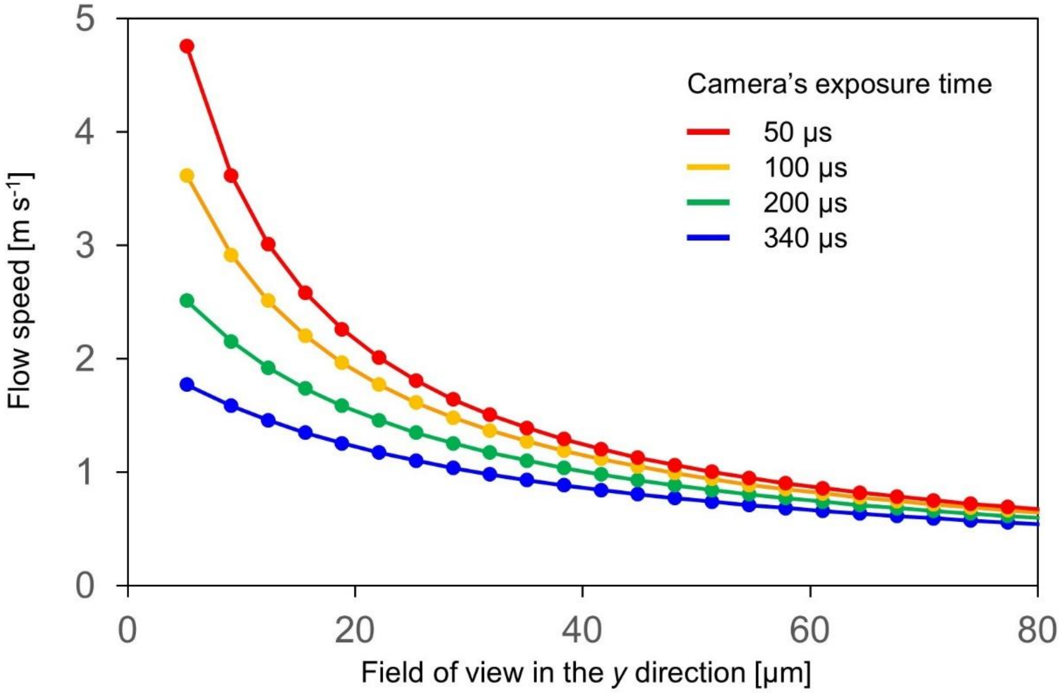

Supplementary Fig. 3 | Relation between the cell flow speed and the FOV in the *y* direction at different camera exposure times. The values were obtained by assuming a magnification of 20 for the imaging system and the use of the sCMOS camera model pco.edge 5.5. The cell flow speed can be increased by decreasing the FOV or shortening the camera exposure time. The source data is available in our Source Data file.

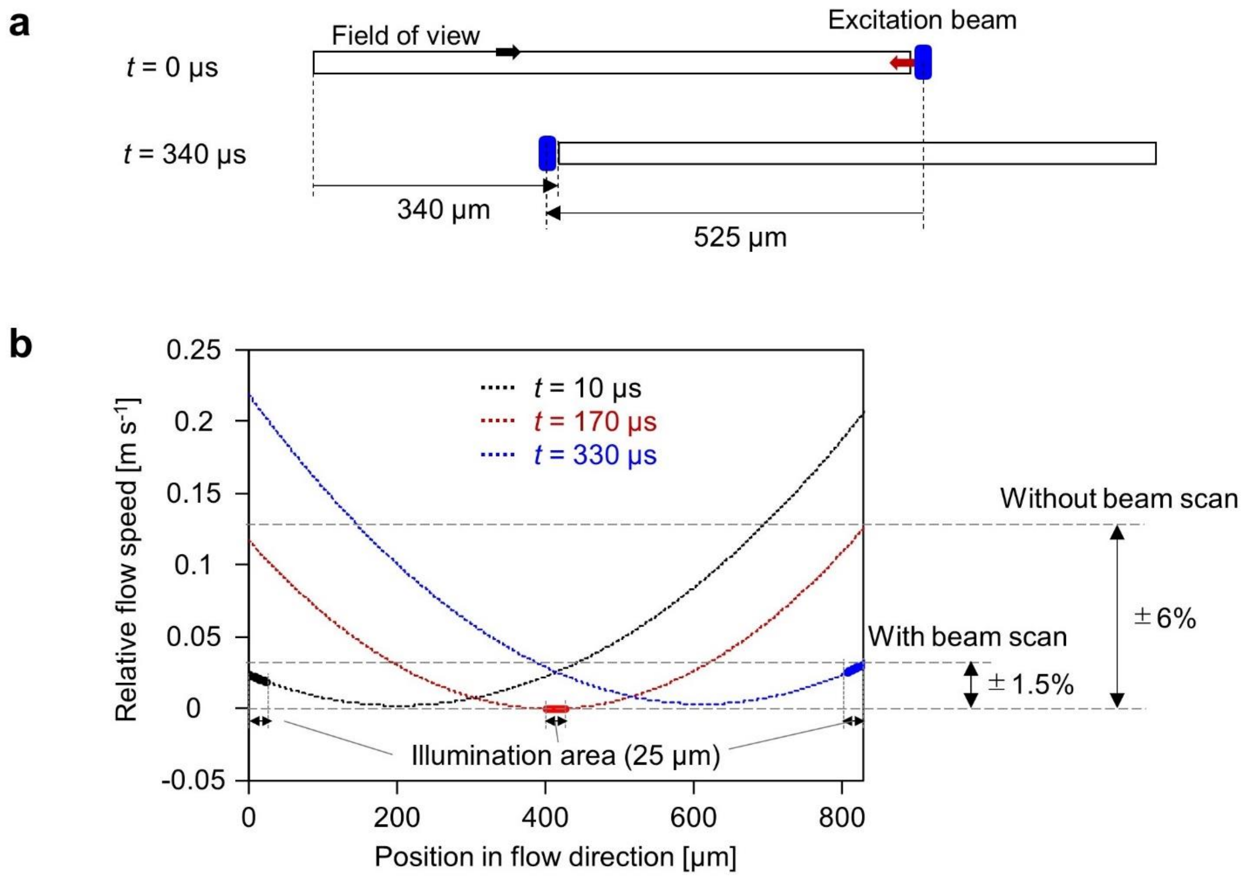

Supplementary Fig. 4 | Details of the excitation beam scan. a, Profile of the excitation beam scan in the object plane. **b,** Residual motion speed of flowing cells with and without the beam scan. The dotted curves represent estimated relative flow speeds at different timings. The solid curves indicate parts of the curves which correspond to illumination areas at the timings.

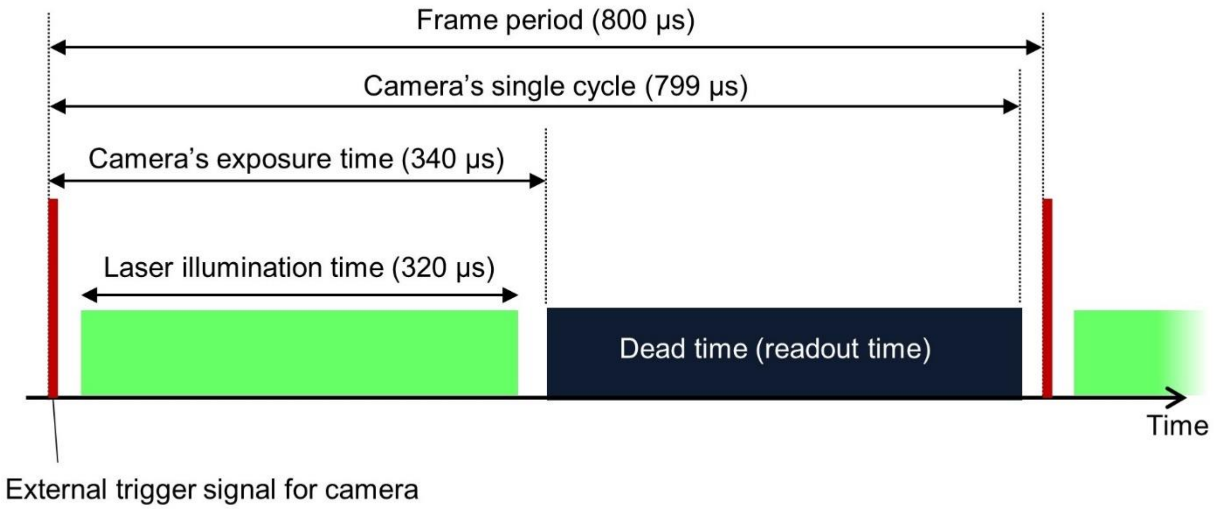

Supplementary Fig. 5 | Schematic of the data acquisition sequence. The sequence of the external triggering of the camera, laser illumination, and image readout repeats at a period of 800 μs. This period is slightly longer than the camera’s single cycle time (time from the external triggering to the end of readout) so that the camera is reliably triggered by every external trigger signal.

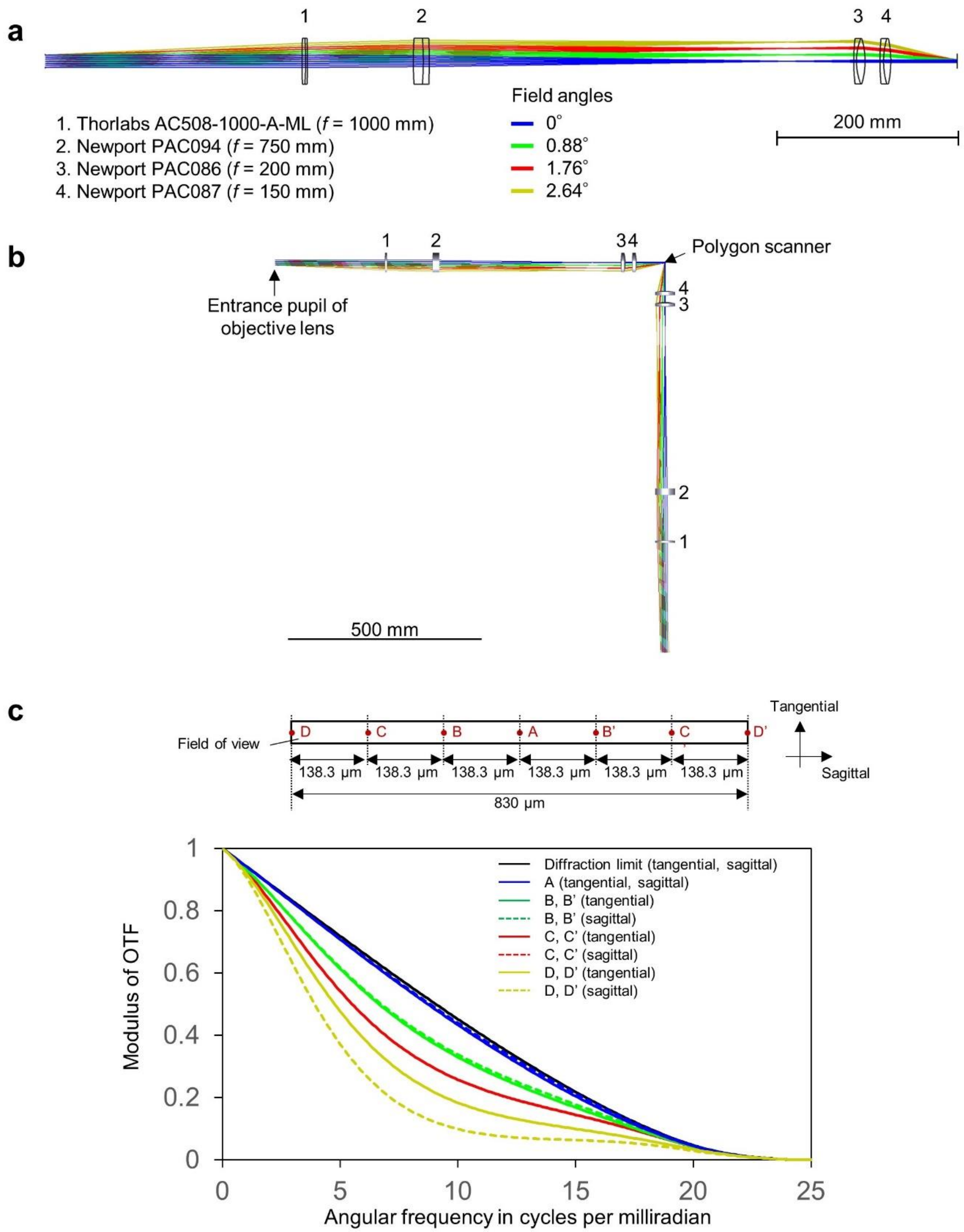

Supplementary Fig. 6 | Design of the relay lens systems. a, Configuration of the designed relay lens system with a magnification of 0.2. **b,** Schematic of the whole relay systems. **c,** OTFs at different positions in the FOV in the object plane.

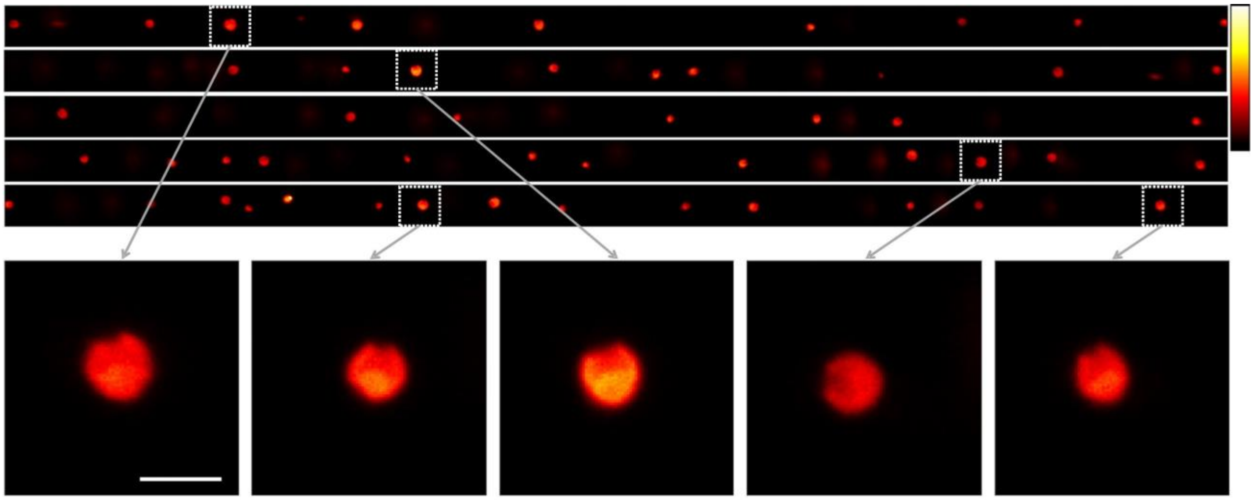

Supplementary Fig. 7 | VIFFI flow cytometry of *Chlamydomonas reinhardtii* cells in a 1-m s^{-1} flow with subcellular resolution and a throughput of ~10,000 cells s^{-1}. An average of ~9 autofluorescence images of *C. reinhardtii* cells were obtained in a single frame (830 μm long in the flow direction with a 30-μm overlapped length between the consecutive frames) at a flow speed of 1 m s^{-1}, demonstrating the fluorescence imaging capability with a throughput of ~10,000 cells s^{-1}. The top five images represent raw frames of the camera with a FOV of 830 μm × 28 μm while the bottom five images show zoomed parts of the frames that show characteristic morphological features (elliptical shape with an indent at the head) of *C. reinhardtii*. A laser source (Oxxius LBX-405-300-CSB-PP, 300 mW, λ = 405 nm) was used as an excitation laser. Scale bar: 10 μm.

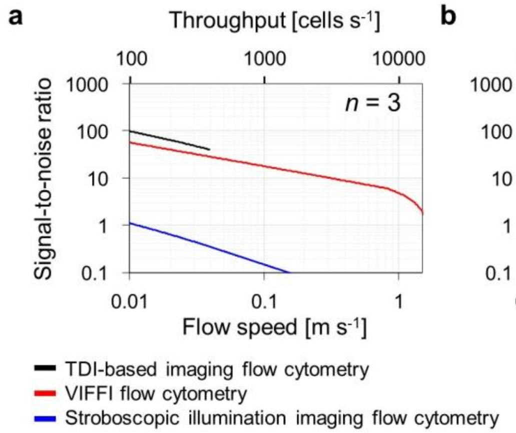

Supplementary Fig. 8 | Estimated SNR for three different methods of IFC. **a**, **b**, and **c** show the SNR at various numbers of fluorescent molecules per pixel (*n*) of 3, 30, and 300, respectively. We assume a pixel size of 0.325 μm and an average cell spacing of 100 μm. VIFFI flow cytometry has a comparable SNR with TDI-based IFC for all different numbers of fluorescent molecules per pixel. The curves of TDI-based IFC end at the flow speed of 0.04 m s^{-1} since it cannot obtain images at >0.04 m s^{-1} due to Amnis ImageStream®^{X} Mark II's upper limit on the line rate. In comparison with stroboscopic illumination IFC, VIFFI flow cytometry provides a significantly higher SNR by more than a factor of 30, enabling image acquisition with a reasonable SNR at a high speed of ~1 m s^{-1}.

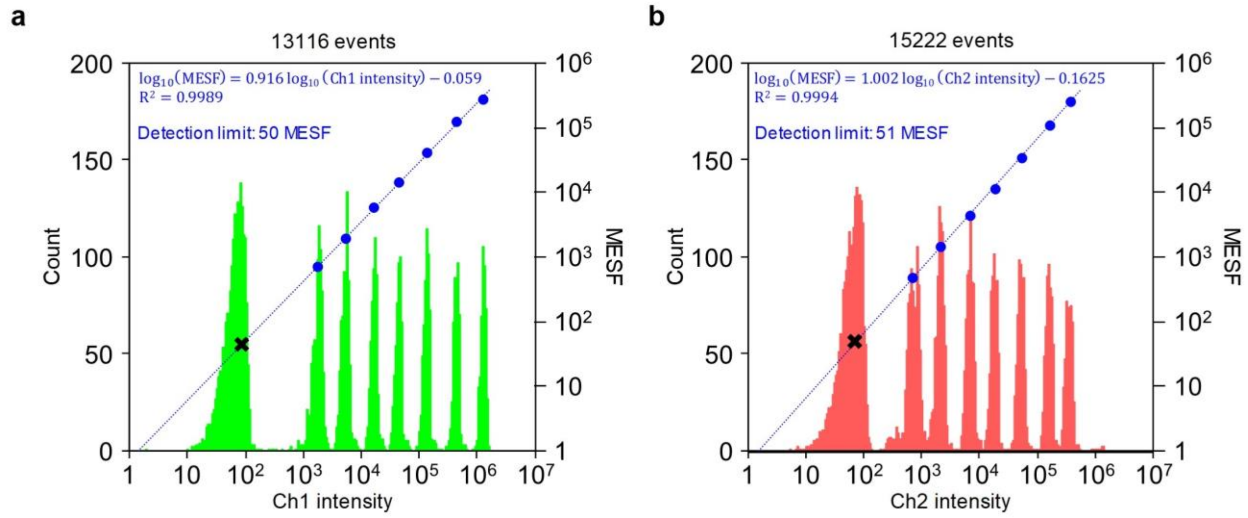

Supplementary Fig. 9 | Evaluation of the detection sensitivity of the VIFFI flow cytometer using standard 8-peak fluorescent calibration particles. a and **b** show results for the green channel (ch1, 510 nm – 580 nm) and the red channel (ch2, > 580 nm), respectively. The histograms show distributions of the signal intensities of the particles with different fluorescence intensities (left vertical axis). We used SPHERO™ Rainbow Calibration Particles (Spherotech Inc., catalog no. RCP-30-5A, lot no. AK02). To detect non-fluorescent particles, we used a microfluidic platform including an additional laser beam (λ = 850 nm) that was detected by a photodetector after passing them through a flow channel. The signal intensities were evaluated as the integrated pixel intensities in circular image areas with a 3.6-μm diameter around the center of the particles (diameter: 3 μm). Defocused images of the particles were excluded from the analysis. The blue dots indicate the average signal intensities and MESF values of the populations (right vertical axis), with regression lines and fit functions shown in blue. The source data is available in our Source Data file.

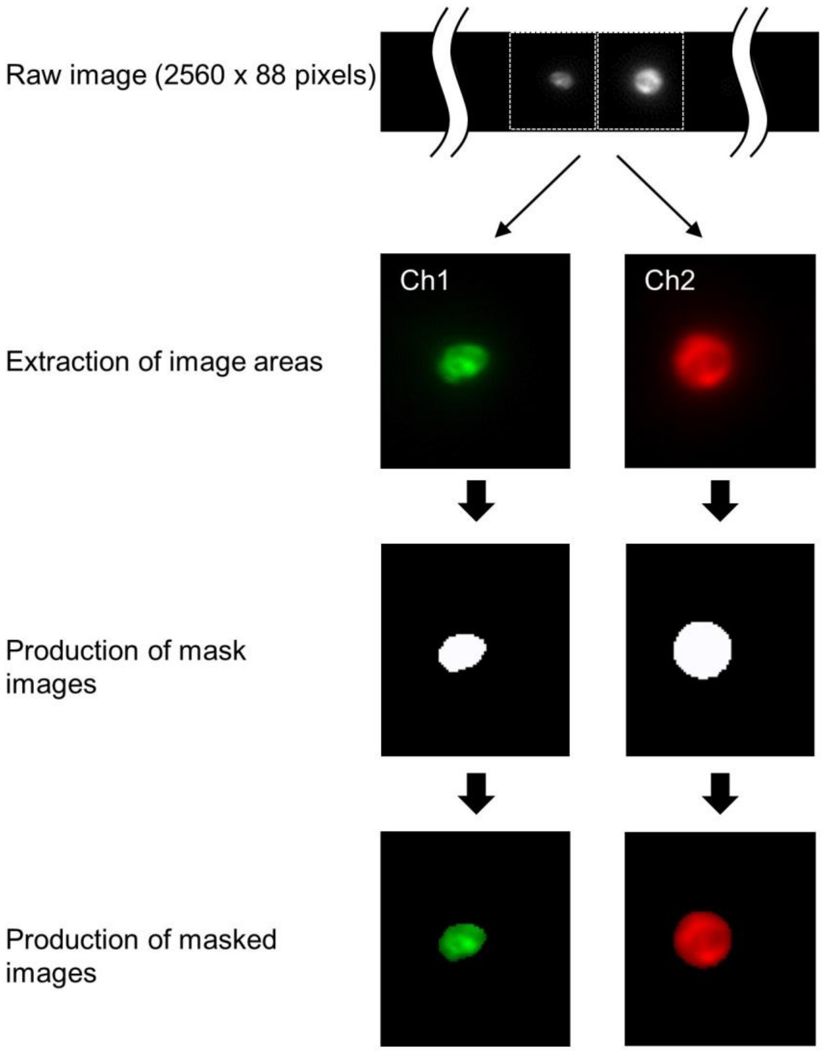

Supplementary Fig. 10 | Flowchart for digital image processing. Two images of a cell that correspond to different color channels are extracted from a raw image. Mask images are created from the extracted image areas, which are used for producing masked cell images.

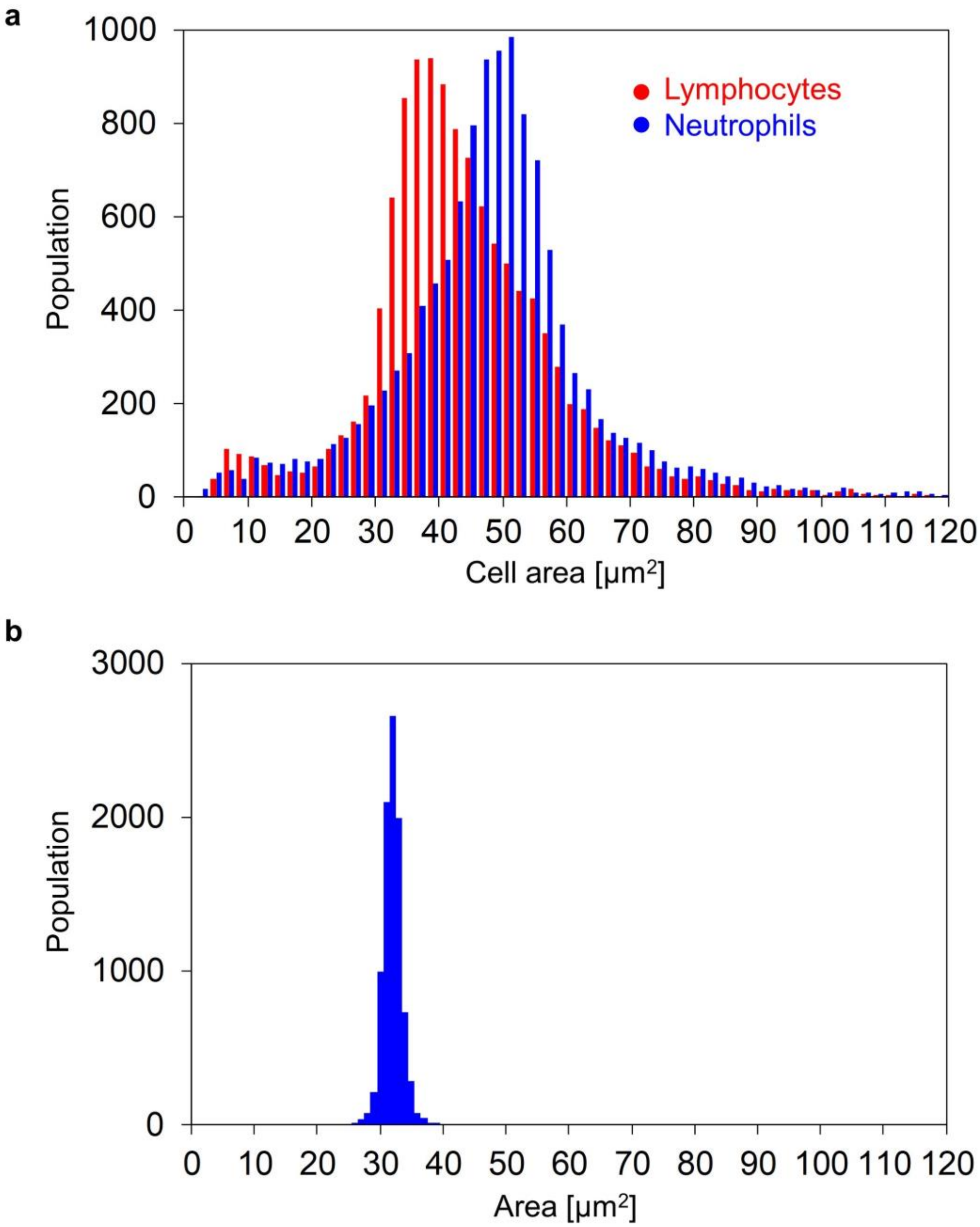

Supplementary Fig. 11 | Statistical analysis of cell areas. a, Histograms of murine lymphocytes and neutrophils in terms of cell area (*n* = 11,930 for lymphocytes and *n* = 12,000 for neutrophils). The source data is available in our Source Data file. **b,** Histogram of 6-μm fluorescent particles in terms of particle area (*n* = 9,253). The narrow distribution of the histogram indicates that the distributions shown in panel **a** reflect those of the cell areas of lymphocytes and neutrophils. We evaluated the area as a masked area with 35% of the peak intensity as a threshold. The source data is available in our Source Data file.

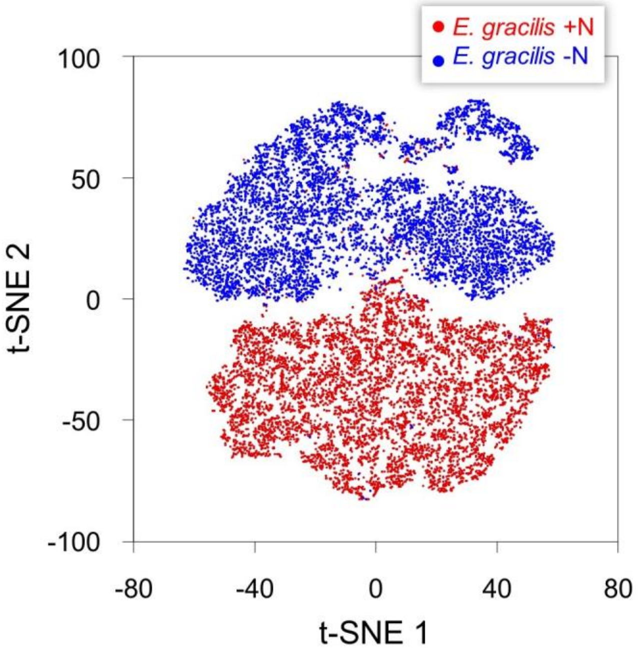

Supplementary Fig. 12 | t-SNE plot of *E. gracilis* cells. Red and blue dots represent *E. gracilis* +N and –N, respectively (n = 8,000 for each group). t-SNE 1 and t-SNE 2 are obtained with 4,096 features by VGG-16. The source data is available in our Source Data file.

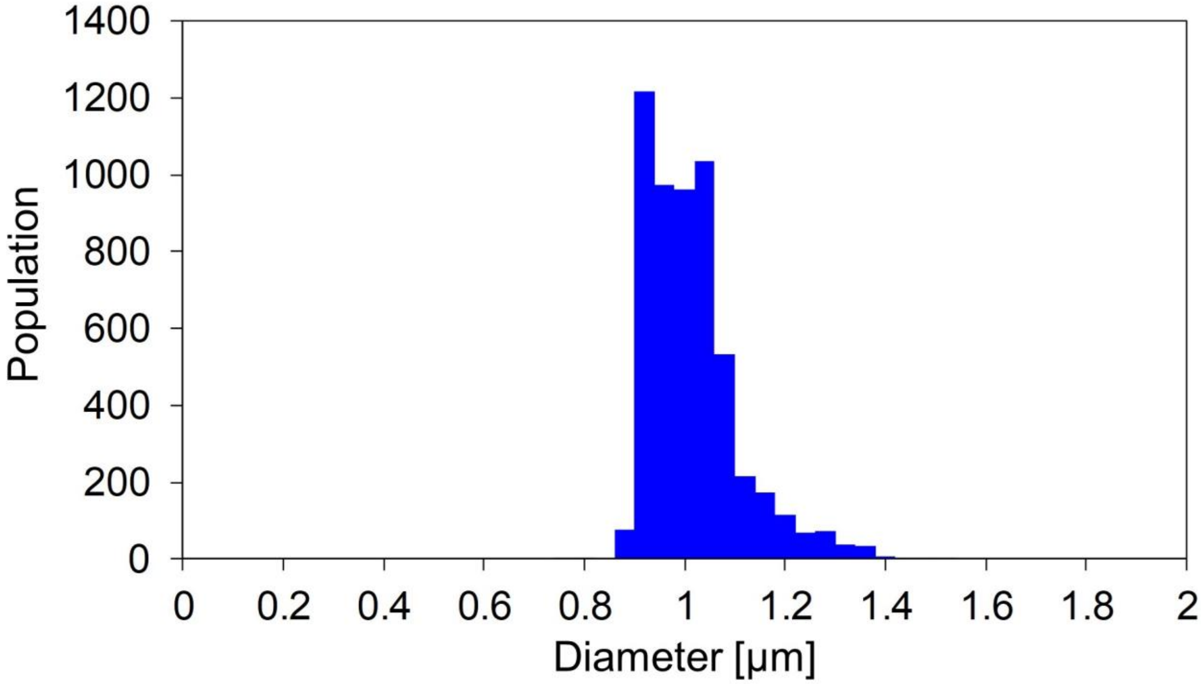

Supplementary Fig. 13 | Histogram of 1-μm fluorescent particles in terms of particle diameter. We evaluated the diameter by FWe^{-1}M of the fitted Gaussian function for the line profile in the y direction (n = 5,539). The tailed shape of the distribution indicates out-of-focus particles. The source data is available in our Source Data file.

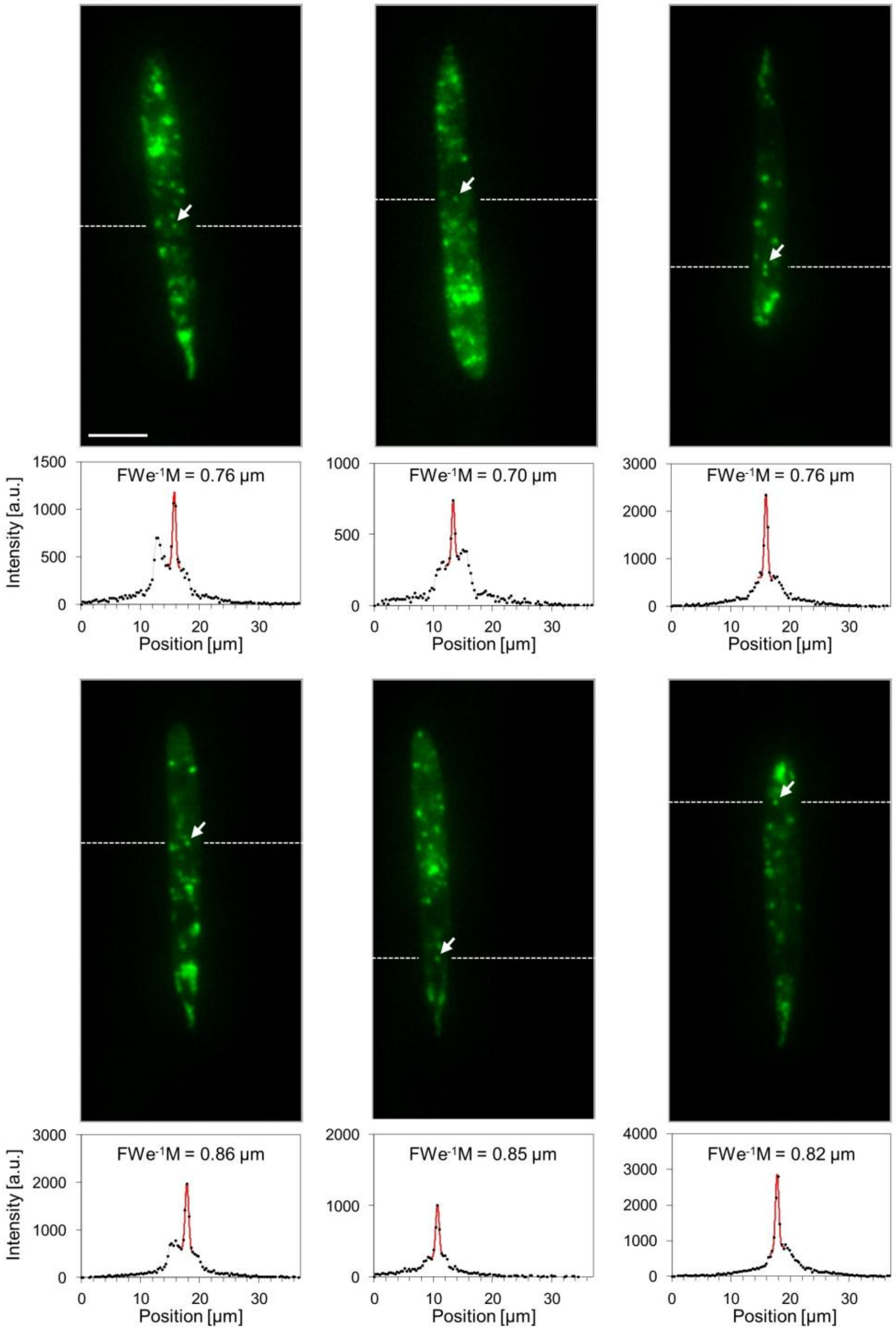

Supplementary Fig. 14 | Evaluation of the size of small lipid droplets in *E. gracilis* cells. The images show lipid droplets in *E. gracilis* cells. The dashed lines correspond to the horizontal axes of the plots below the images. The arrows in the images indicate lipid droplets whose diameters are evaluated in the plots. The black dots connected by the gray lines indicate pixel values. The red curves indicate fitting results for the peaks of small droplets with a Gaussian function with a slanted (linearly varying) background. The source data is available in our Source Data file. Scale bar: 10 µm.

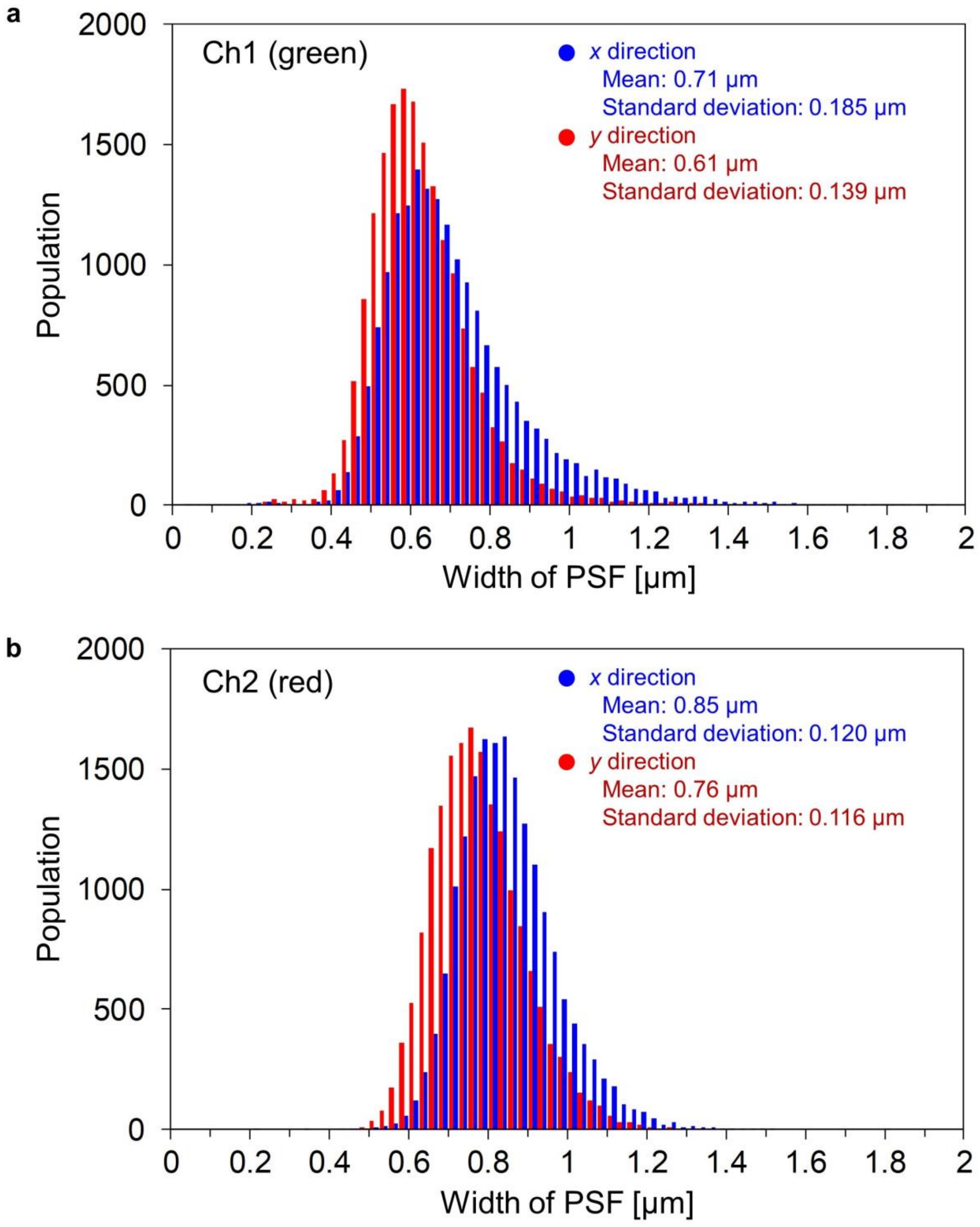

Supplementary Fig. 15 | Statistical analysis of the spatial resolution. a, Histograms of $FWe^{-1}M$ PSF widths in the green channel (ch1, 510 nm – 580 nm) in the *x* and *y* directions. **b,** Histograms of $FWe^{-1}M$ PSF widths in the red channel (ch2, > 580 nm) in the *x* and *y* directions. All the histograms were obtained from a single set of images of 200-nm fluorescent beads (*n* = 18,000). The source data is available in our Source Data file.

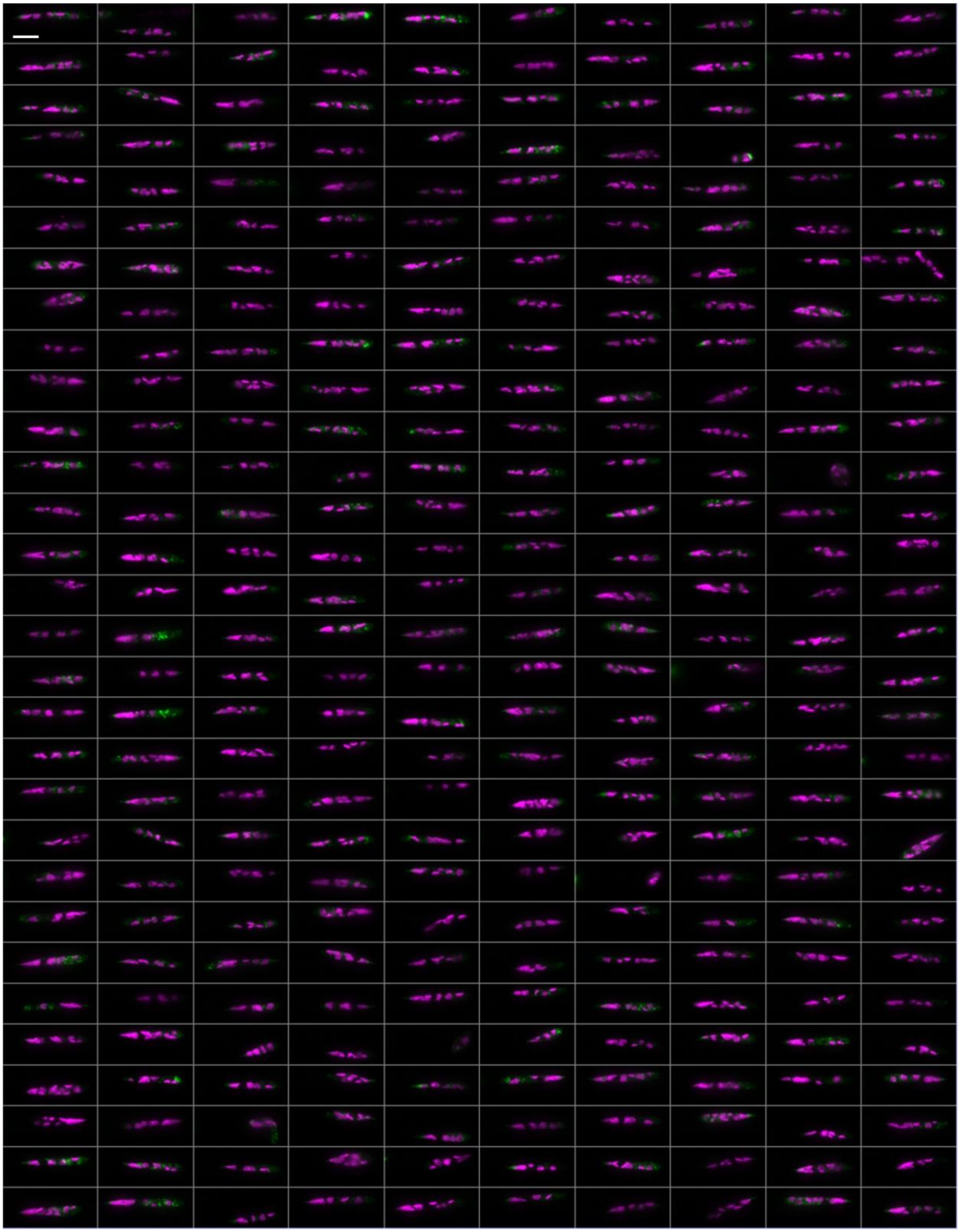

Supplementary Fig. 16 | Images of *E. gracilis* +N cells obtained by the VIFFI flow cytometer. The lipids (shown in green) were stained by BODIPY505/515 while chlorophyll (shown in magenta) was autofluorescent. Scale bar: 20 µm.

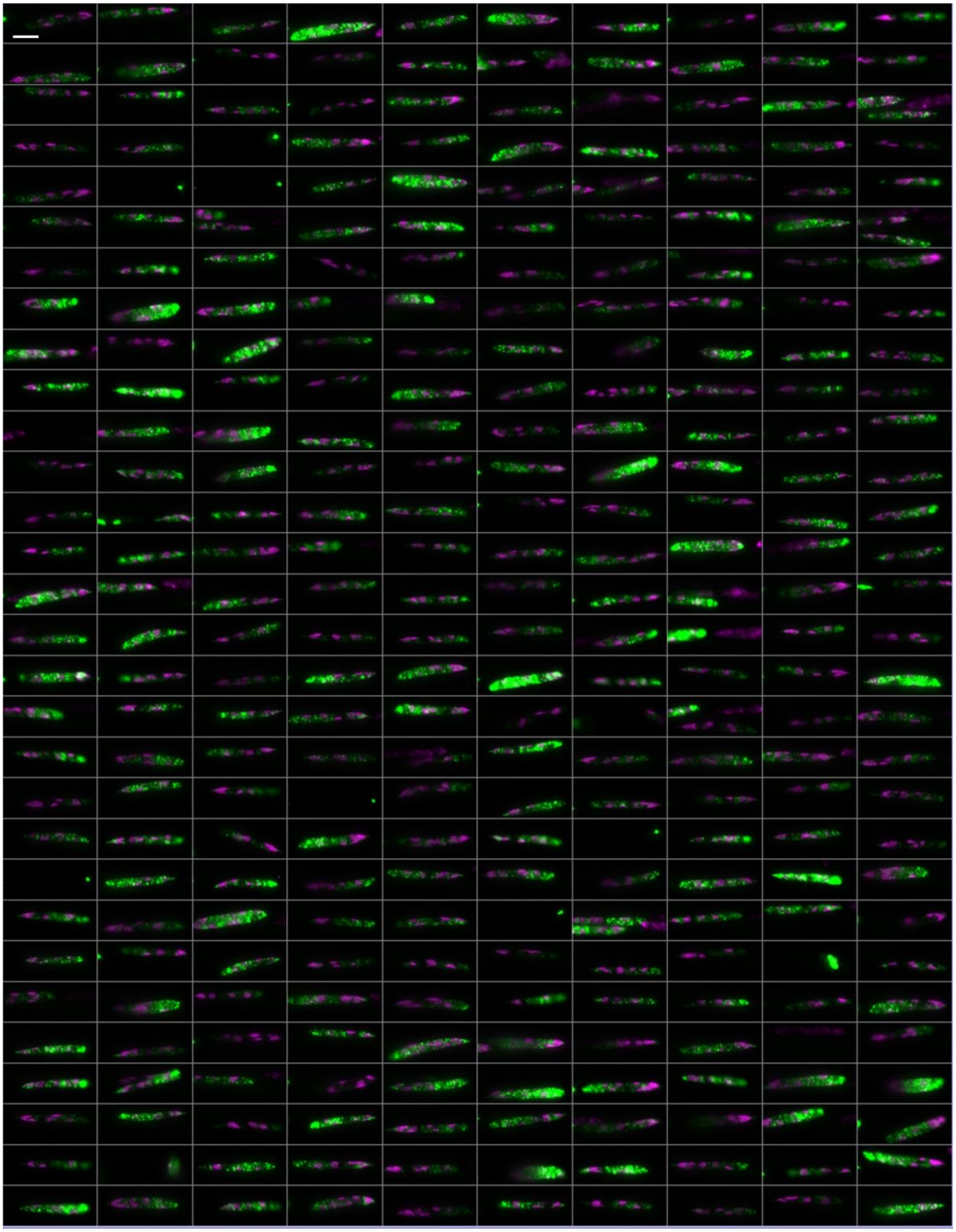

Supplementary Fig. 17 | Images of *E. gracilis* –N cells obtained by the VIFFI flow cytometer. The lipids (shown in green) were stained by BODIPY505/515 while chlorophyll (shown in magenta) was autofluorescent. Scale bar: 20 µm.

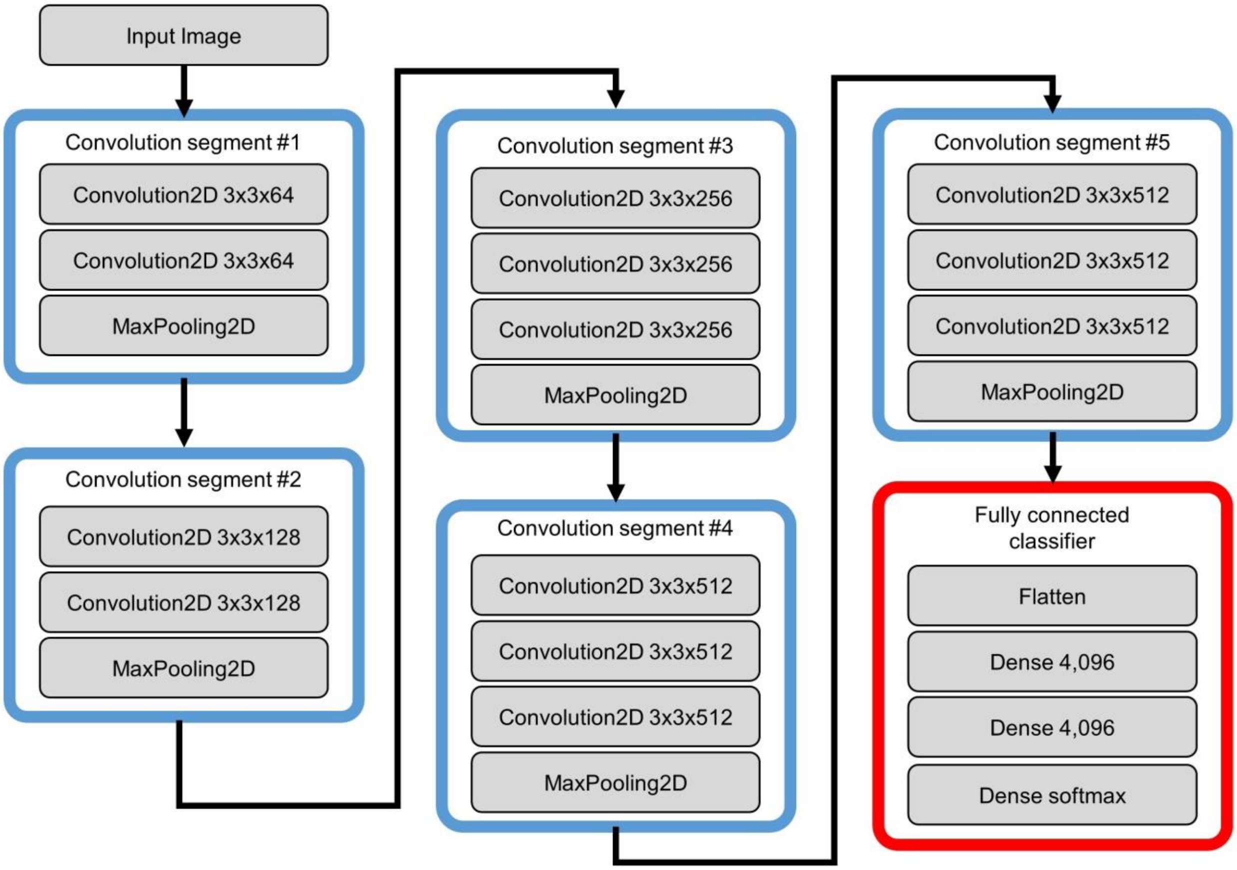

Supplementary Fig. 18 | Network architecture of VGG16. 4,096 features are extracted from a (multi-color) input image through five convolution segments and a fully connected classifier. Each convolution segment consists of two or three convolutional layers for feature extraction and a maximum pooling layer for reduction of data volume. The fully connected classifier downgrades the 4,096 features to one dimension to provide a classification result.

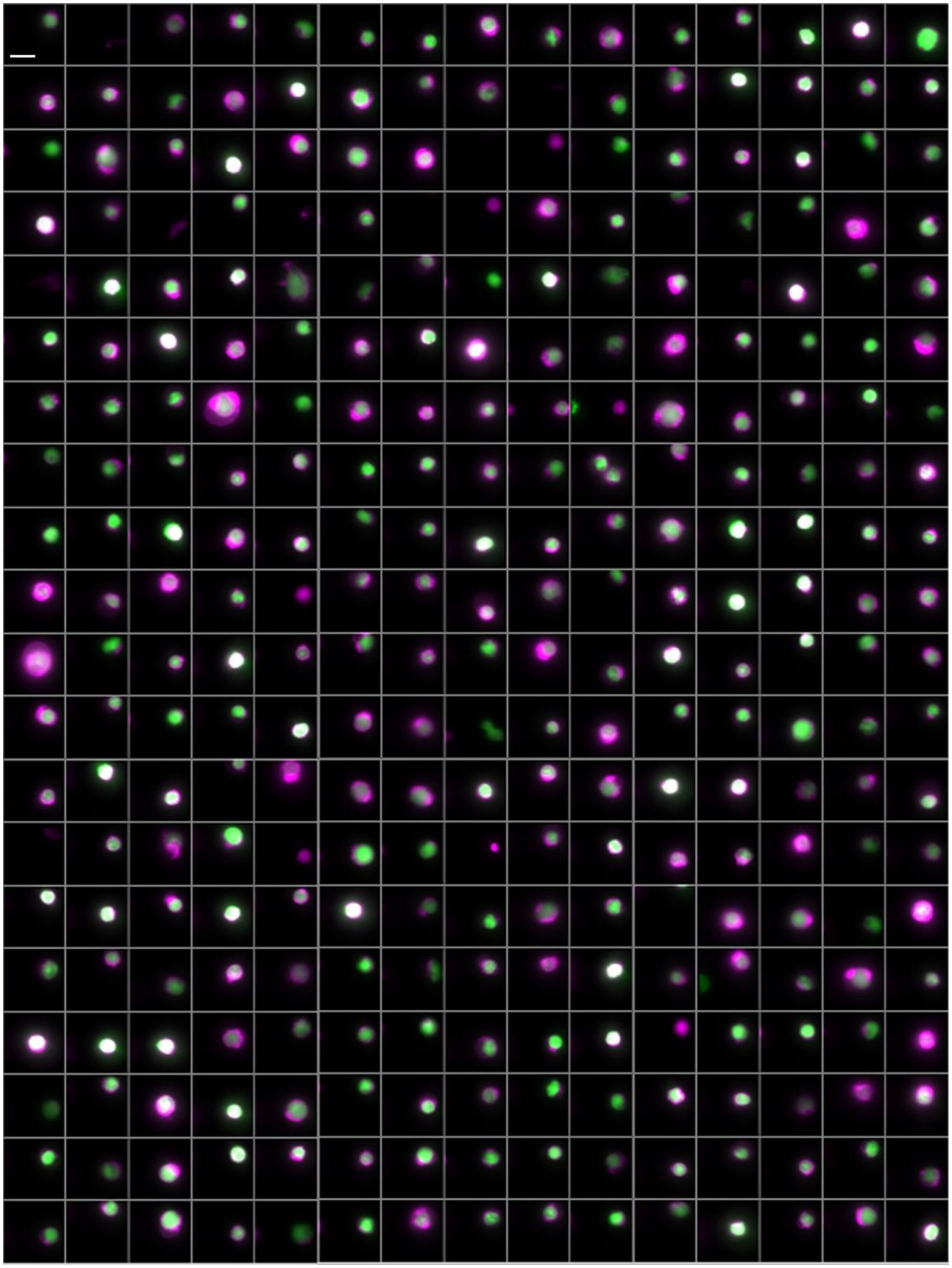

Supplementary Fig. 19 | Images of murine lymphocytes obtained by the VIFFI flow cytometer. The cytoplasm was stained by CellTracker Red (shown in magenta) while the nucleus was stained by SYTO16 (shown in green). Scale bar: 10 µm.

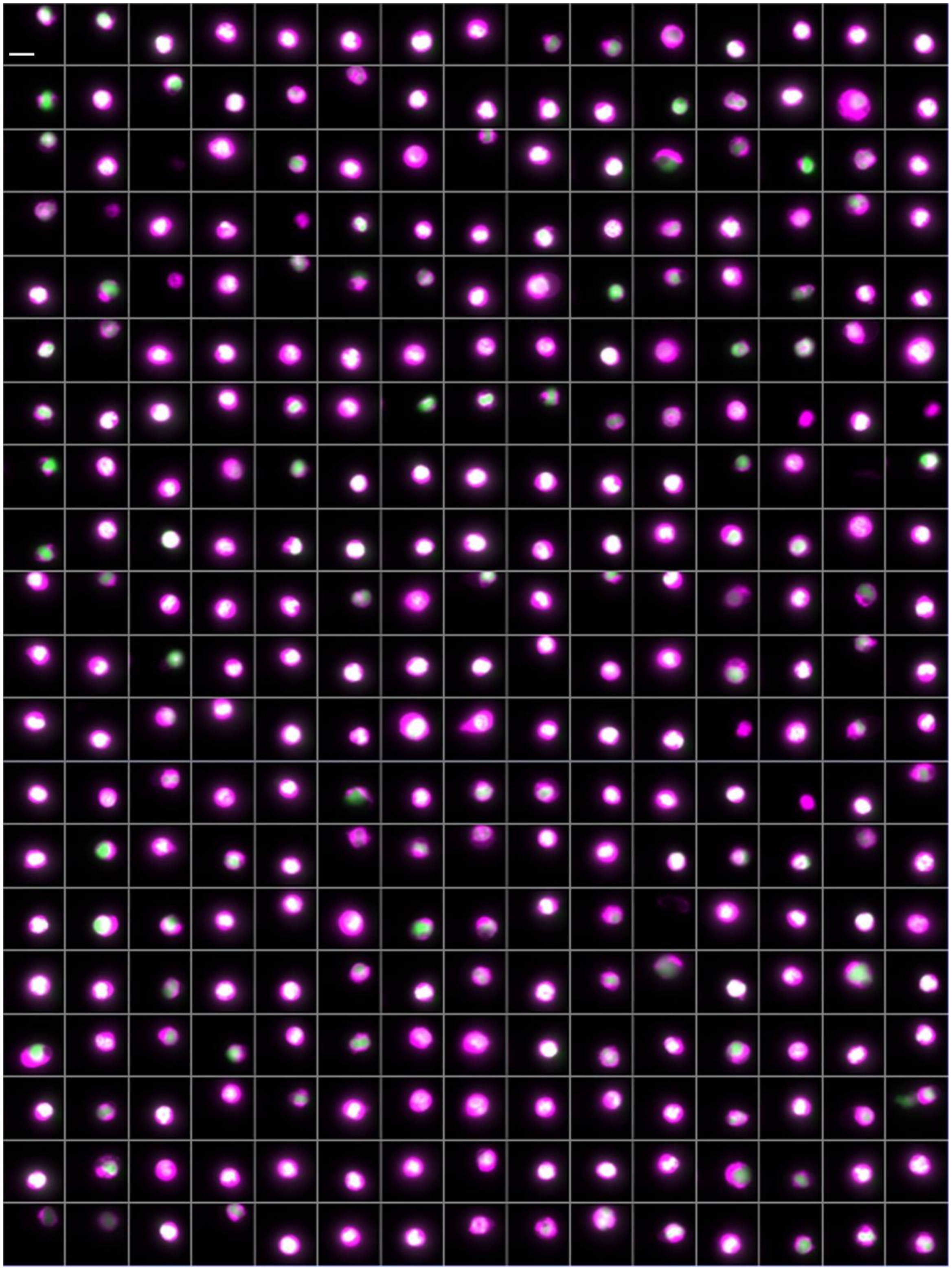

Supplementary Fig. 20 | Images of murine neutrophils obtained by the VIFFI flow cytometer. The cytoplasm was stained by CellTracker Red (shown in magenta) while the nucleus was stained by SYTO16 (shown in green). Scale bar: 10 µm.

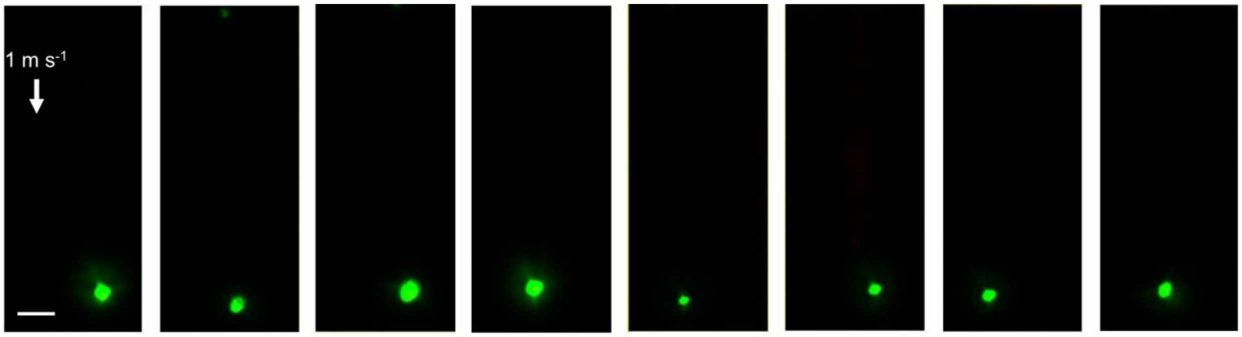

Supplementary Fig. 21 | Images of isolated lipid droplets obtained by the VIFFI flow cytometer. These images correspond to the near-origin peak of the *E. gracilis* –N histogram in Fig. 4c. Scale bar: 10 µm.

Supplementary Table 1 | Parameter settings for the estimation of the SNRs.

		VIFFI flow cytometry		TDI-based IFC		Stroboscopic illumination IFC	
Parameter	Symbol	Value	Note	Value	Note	Value	Note
Excitation beam power [mW]	*P*	180	Accounting for ~10% loss at the beam scanner	200	Maximum output power of the 488-nm laser in ImageStream®^{X} Mark II	200	Same as the left for fair comparison
Excitation beam diameter in the depth direction [μm]	D_z	4	Current setting	4	Same as the left value for fair comparison	4	Same as the left value for fair comparison
Excitation beam diameter in the flow direction [μm]	D_x	26	Current setting	26	Same as the left value for fair comparison	666	Full FOV in the flow direction at 20x
Cross section of the excitation beam [μm^2]	*C*	104	Approximated by $\pi D_x D_z/4$	104	Approximated by $\pi D_x D_z/4$	104	Approximated by $\pi D_x D_z/4$
Time of the excitation beam illumination	*T*	(vari-able)	$\min(t_{exp1}, t_{exp2})D_x/FOV_x$ (see Eqs. S5 and S6)	(vari-able)	D_x/(flow speed)	(vari-able)	(pixel size)/(flow speed)
Absorption cross section [cm^2]	*s*	1.16×10^{-16}	Fluorescein	1.16×10^{-16}	Fluorescein	1.16×10^{-16}	Fluorescein
Quantum yield	η_{yield}	0.8	Fluorescein	0.8	Fluorescein	0.8	Fluorescein
Photon collection efficiency of the imaging system	η_{opt}	0.0277	Calibrated value of the present setup at NA = 0.75	0.0305	10% higher than the left value, NA = 0.75	0.0129	Same as the left at the same NA, NA = 0.5
Quantum efficiency of the image sensor	η_{sensor}	0.55	Specification of pco.edge 5.5 (λ = 530 nm)	0.8	Specification of Hamamatsu C10000-801 (λ = 530 nm)	0.83	Specification of Hamamatsu ORCA-Flash 4.0 V3 (λ = 530 nm)
Readout noise [e$^-$ rms]	σ	1.7	Specification of pco.edge 5.5 (λ = 530 nm)	50	Specification of Hamamatsu C10000-801 (λ = 530 nm)	1.6	Specification of Hamamatsu ORCA-Flash 4.0 V3 (λ = 530 nm)

Italic characters represent variables.

Supplementary Table 2 | Comparison of parameters related to data acquisition speed.

	VIFFI flow cytometry	ImageStream®^{X} Mark II
Pixel size	0.325 μm	0.33 μm[1)]
Flow speed	1 m s^{-1}	0.04 m s^{-1} [2)]
Effective line rate	3.1 MHz	0.12 MHz
Cell throughput at an average cell spacing of 100 μm	10,000 cells s^{-1}	400 cells s^{-1}

1) Specification of ImageStream®^{X} Mark II at 60-X magnification

2) INSPIRE® (ImageStreamX® System Software) User's Manual Version Mark II
