# Supplementary material for: Virtual-freezing fluorescence imaging flow cytometry
Source: Nat Commun. 2020 Mar 6;11:1162. doi: 10.1038/s41467-020-14929-2 (PMC7058616; doi:10.1038/s41467-020-14929-2)
Supplement: Supplementary file 3 — Description of Additional Supplementary Files [file 41467_2020_14929_MOESM3_ESM.pdf]

Description of Additional Supplementary Files

File Name: Supplementary Movie 1
Description: Principles of VIFFI flow cytometry.
